# Why do we Need Quality Articles, What are we Going to do With?

**DOI:** 10.4103/0975-1483.80289

**Published:** 2011

**Authors:** KK Mueen Ahmed

**Affiliations:** *Editor, J Young Pharm. E-mail: mueen.ahmed@gmail.com*

I welcome you to this second issue for the year 2011 comprising very interesting articles to read. We are proud to inform about the inclusion of our journal in SCImago Journal Ranking [SJR] recently. SJR is one of the two major journal rating systems that exist in the world: the Journal of Citation reports by ISI-Thomson and the SCImago indexes issued by Elsevier. The SCImago Journal and Country Rank portal is developed by SCImago Research Group working at three Spanish universities (Consejo Superior de Investigaciones Científicas (CSIC), University of Granada, Extremadura, Carlos III (Madrid) and Alcalá de Henares). It is named after the SCImago Journal Rank Indicator (SJR) developed by the group. The citation data used is derived from Scopus database and journal rankings are available for journals contained in the Scopus database. The SJR indicator is calculated based on three year’s citation data and attributes different weight to citations depending on the prestige of the citing journal. The prestige of a journal is estimated using PageRank algorithm in the network of journals. The prestige of a journal is transferred through the references that a journal receives from other journals.[1–3]

SJR began publishing **Cites/Document (C/D)** for its own collection of journals. The following calculation used to determine impact factors [IFs] and Cites/Document [C/D]. Briefly, the IF or the C/D for any journal “J” in any year “N” is given by the following equation:

$${\mathrm{IF}}_{\left(J,N\right)}\;=\;C/D_{\left(J,N\right)}\;=\;C_{\left(N\right)}/\left(A_{\left(N-2\right)}+A_{\left(N-1\right)}\right),$$

where *C*(*N*) is the total number of cites appearing in journals from each respective collection to articles published by journal “*J*” in years “*N*-2” and “*N*-1”, and *A(N-i)* is the number of articles published by “*J*” in years “*N*-1" and “*N*-2”. It will help us to analyse the journal metrics of Journal of Young Pharmacists, based on our citation performance. By end of this year, we are very much expecting a good citation scale for our journal. This issue brings you many articles covering in different field of pharmaceutical sciences.

## PEER REVIEW VS. ARTICLE QUALITY

Peer review is a process carried out by experts in a related field of article subject and presents their opinions in order to check and validate the research being done and submitted to a journal. We have a good number of reviewers assisting us in refining the manuscript and improving the article quality based on their suggestions. However, this process is always a tussle, sometimes time-based, or response-based. We are identifying and adding the reviewers at regular basis. We thank all the active reviewers of J Young Pharm for their quality review, which helped us getting good indexing status in a short span of time. Journalonweb.com[4] (Courtesy: Medknow) has made this process quite easier and simple and the process is totally paper free and works electronically. We strive hard to speed up this process to comfort our authors and reducing their waiting time.

## JOURNAL QUALITY

An average 15 to 16 articles in each issue takes us 3–6 months to complete the peer review process and refining the quality of papers. Present days have seen many journals, which are publishing nearly hundreds of manuscripts in a single issue; it is highly impossible to conduct peer review process in a quarter, it is being worst part of the journal publishing and such journals will damage the country’s image on scholarly content and its publishing. Authors should be wise enough and have good sense in choosing a right journal, which will impact their writing and could be further used and cited by other researchers. Journal quality will always be judged based on many factors, such as editor’s experience, Editorial board, Quality of articles, Quality of peer review, Reviewers, publishing on time, non-interruption in publishing of issues etc.[5,6] Authors should not just focus on just adding on number of papers in their resume, instead they should start adding H-index and IF value of their published papers.

## SUBMISSION FOCUS

At present, J Young Pharm has seen many submissions in different fields of pharmaceutical sciences, as we have limited room to include articles in each category. I recommend few relevant submissions could be merely focused on specific subjects, as we have many associated journals providing the same quality. You can also consider submitting review articles in Systematic Reviews in Pharmacy [www.sysrevpharm.org],[7] pharmacology-based papers in Journal of Pharmacology and Pharmacotherapeutics [www.jpharmacol.com],[8] analysis-based papers in Pharmaceutical Methods [www.phmethods.org],[9] pharmaceutics-based papers in International Journal of Pharmaceutical Investigation [www.jpionline.org].[10]

Finally, I would like to acknowledge with gratitude the support of Medknow Publications and Media Pvt. Ltd, Mumbai, India. We are happy to hear, any criticism, suggestions and appreciations. Please feel free to write to us at any point of time during your publication process.

Happy reading!!!
